# *catena*-Poly[[[aqua­(2,2′-bipyridine)manganese(II)]-μ-5-methoxy­iso­phthalato-κ^3^ *O*,*O*′:*O*′′] monohydrate]

**DOI:** 10.1107/S1600536809035466

**Published:** 2009-09-05

**Authors:** Su-Mei Shen

**Affiliations:** a Department of Applied Engineering, Zhejiang Economic and Trade Polytechnic, 310018 Hangzhou, People’s Republic of China

## Abstract

In the title compound, {[Mn(C_8_H_4_O_4_)(C_10_H_8_N_2_)(H_2_O)]·H_2_O}_*n*_, the Mn^II^ centre is octa­hedrally coordinated by three O atoms from two 5-methoxy­isophthalate (CH_3_O-ip) ligands, a fourth from a coordinated water mol­ecule and two N atoms from one chelating 2,2′-bipyridine (2,2-bipy) ligand. Each pair of adjacent Mn^II^ atoms is bridged by a CH_3_O-ip ligand, forming a helical chain running along a crystallographic 2_1_ axis in the *c*-axis direction. These chains are decorated with 2,2′-bipy ligands on alternating sides. O—H⋯O hydrogen bonding involving the water molecules stabilizes the crystal structure.

## Related literature

For related structures, see: Chen & Liu, (2002 ▶); Liu *et al.* (2009 ▶). For the design and controlled synthesis of metal-organic frameworks, see: Kitagawa *et al.* (2004 ▶). For the use of 5-methoxy­isophthalic acid in synthesis of self-asssembly of porous coord­in­ation compounds, see: Ma *et al.* (2009 ▶).
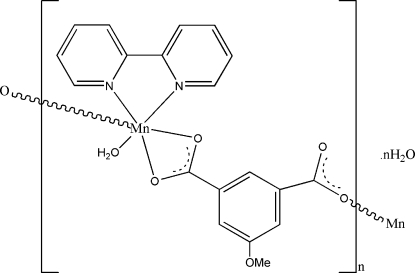


## Experimental

### 

#### Crystal data

[Mn(C_8_H_4_O_4_)(C_10_H_8_N_2_)(H_2_O)]·H_2_O
*M* *_r_* = 441.29
Monoclinic, 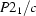

*a* = 8.9067 (13) Å
*b* = 17.367 (3) Å
*c* = 12.5804 (18) Å
β = 97.176 (2)°
*V* = 1930.7 (5) Å^3^
*Z* = 4
Mo *K*α radiation
μ = 0.73 mm^−1^
*T* = 298 K
0.19 × 0.14 × 0.09 mm

#### Data collection

Bruker APEXII area-detector diffractometer
Absorption correction: multi-scan (*SADABS*; Bruker, 2005 ▶) *T*_min_ = 0.874, *T*_max_ = 0.937
11370 measured reflections
3470 independent reflections
2275 reflections with *I* > 2σ(*I*)
*R*_int_ = 0.061

#### Refinement

*R*[*F* ^2^ > 2σ(*F* ^2^)] = 0.044
*wR*(*F* ^2^) = 0.091
*S* = 1.02
3470 reflections
275 parameters
6 restraints
H atoms treated by a mixture of independent and constrained refinement
Δρ_max_ = 0.26 e Å^−3^
Δρ_min_ = −0.29 e Å^−3^

### 

Data collection: *APEX2* (Bruker, 2005 ▶); cell refinement: *SAINT* (Bruker, 2005 ▶); data reduction: *SAINT* (Bruker, 2005 ▶); program(s) used to solve structure: *SHELXS97* (Sheldrick, 2008 ▶); program(s) used to refine structure: *SHELXL97* (Sheldrick, 2008 ▶); molecular graphics: *ORTEPIII* (Burnett & Johnson, 1996 ▶), *ORTEP-3 for Windows* (Farrugia, 1997 ▶) and *PLATON* (Spek, 2009 ▶); software used to prepare material for publication: *SHELXL97*.

## Supplementary Material

Crystal structure: contains datablocks I, global. DOI: 10.1107/S1600536809035466/bg2299sup1.cif

Structure factors: contains datablocks I. DOI: 10.1107/S1600536809035466/bg2299Isup2.hkl

Additional supplementary materials:  crystallographic information; 3D view; checkCIF report

## Figures and Tables

**Table 1:** Hydrogen-bond geometry (Å, °)

*D*—H⋯*A*	*D*—H	H⋯*A*	*D*⋯*A*	*D*—H⋯*A*
O6—H1*W*⋯O7^i^	0.837 (17)	1.832 (18)	2.668 (4)	178 (4)
O6—H2*W*⋯O3^ii^	0.846 (17)	1.888 (19)	2.726 (3)	171 (3)
O7—H3*W*⋯O2^iii^	0.839 (18)	1.90 (2)	2.724 (3)	169 (4)

Symmetry codes: (i) 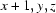; (ii) 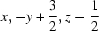; (iii) 
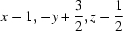
.

**Fig. 1.**
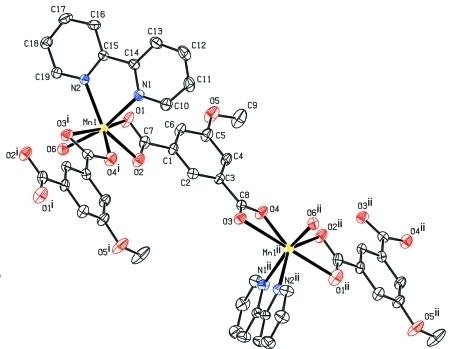
The ORTEP plot of (I), showing the atom-labeling scheme. Ellipsoids are drawn at the the 30% probability level. Symmetry codes: (i) 2-x, y-1/2, 3/2-z; (ii) 2-x, y+1/2, 3/2-z

**Fig. 2.**
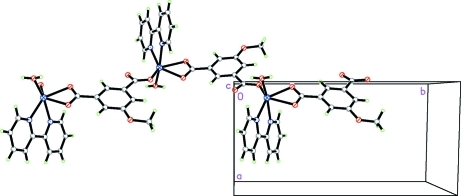
A partial packing virçew of the title compounds, showing the formation of a chain along c axis.

## supplementary crystallographic information

### Comment

Much effort has been focused on the design and controlled synthesis of metal-organic frameworks (Kitagawa *et al.*, 2004). Polycarboxylate ligands have received considerable attention, owing to the variety of their coordination modes and structural features. 5-Methoxyisophthalic acid is a potential multi-dentate ligand with a versatile coordination mode, which has been used in self-asssembled porous coordination synthesis (Ma *et al.*, 2009). The title compound, (I), was constructed by two kinds of bridging and chelating ligands under mild condition, CH_3_O-ip and 2,2'-bipy which were self-assembled to a one-dimensional neutral metal-organic compound. In this paper, the crystal structure of (I) is presented.

As illustrated in Fig. 1, Mn^II^ adopts a distorted octahedral geometry, generated by three O atoms from one bidenated-chelating carboxylate and one monodenated carboxylate group from two adjacent CH_3_O-ip, a fourth O from a coordinated water molecule, and two N atoms from one chelating 2,2-bipy ligand. The four atoms (O1, O2, O4 and N2) in the equatorial plane around the Mn atom form a highly distorted square-planar arrangement, while the distorted octahedral coordination is completed by the N atom of 2,2-bipy (N1) and the O atom of the water molecule (O6) in the axial positions.

The neighboring Mn atoms are linked by CH_3_O-ip ligands forming a one-dimensional helical chain running along a crystallographic 2_1_ axis in the c-direction (Fig. 2). These chains are decorated with 2,2'-bipy ligands alternating at both sides, which is similar to some already reported complexes (Chen & Liu, 2002; Liu *et al.*, 2009 ). There are no remarkable π-π interactions between rings of 2,2'-bipy ligands due to its transplacement arrange.

In the crystal structure, strong intermolecular O-H···O hydrogen bonds (Table 2) link the molecules into a 2D network.

### Experimental

The title compound was obtained by direct mixing of equimolar (21mg, 0.1mmol)) Mn(AC)_2_  water solution (4mL) and CH_3_O-H_2_ip (20mg, 0.1mmol), 2,2'-bipy (0.19mg, 0.1mmol) and NaOH (3.8mg, 0.09mmol) 96% methanol solutions (10mL). After a few days, some crystalline material had precipitated, but it was found to be unsuitable for X-ray diffraction. This material was therefore dissolved in water and heated at 398 K for 3 h in a pressurized reactor. Slow evaporation of this solution resulted in the formation of some block crystals of (I), which were suitable for X-ray analysis.

### Refinement

All H atoms attached to C atoms were placed geometrically and treated as riding with C—H = 0.93 Å (aromatic) with U_iso_(H) = 1.2U_eq_(C). H atoms of water molecule were located in difference Fourier maps and included in the subsequent refinement using restraints (O-H= 0.85 (2)Å and H···H= 1.38 (2)Å) with U_iso_(H) = 1.5U_eq_(O). The highest residual difference electron-density peak occurs close to atom O1 with 1.17Å.

### Crystal data

**Table:**

[Mn(C_8_H_4_O_4_)(C_10_H_8_N_2_)(H_2_O)]·H_2_O	*F*(000) = 908
*M**_r_* = 441.29	*D*_x_ = 1.518 Mg m^−^^3^
Monoclinic, *P*2_1_/*c*	Mo *K*α radiation, λ = 0.71073 Å
Hall symbol: -P 2ybc	Cell parameters from 3462 reflections
*a* = 8.9067 (13) Å	θ = 2.6–25.2°
*b* = 17.367 (3) Å	µ = 0.73 mm^−^^1^
*c* = 12.5804 (18) Å	*T* = 298 K
β = 97.176 (2)°	Block, yellow
*V* = 1930.7 (5) Å^3^	0.19 × 0.14 × 0.09 mm
*Z* = 4	

### Data collection

**Table:**

Bruker APEXII area-detector diffractometer	3470 independent reflections
Radiation source: fine-focus sealed tube	2275 reflections with *I* > 2σ(*I*)
graphite	*R*_int_ = 0.061
φ and ω scans	θ_max_ = 25.2°, θ_min_ = 2.6°
Absorption correction: multi-scan (*SADABS*; Bruker, 2005)	*h* = −10→10
*T*_min_ = 0.874, *T*_max_ = 0.937	*k* = −20→20
11370 measured reflections	*l* = −14→14

### Refinement

**Table:**

Refinement on *F*^2^	Primary atom site location: structure-invariant direct methods
Least-squares matrix: full	Secondary atom site location: difference Fourier map
*R*[*F*^2^ > 2σ(*F*^2^)] = 0.044	Hydrogen site location: inferred from neighbouring sites
*wR*(*F*^2^) = 0.091	H atoms treated by a mixture of independent and constrained refinement
*S* = 1.02	*w* = 1/[σ^2^(*F*_o_^2^) + (0.0276*P*)^2^ + 0.6024*P*] where *P* = (*F*_o_^2^ + 2*F*_c_^2^)/3
3470 reflections	(Δ/σ)_max_ = 0.001
275 parameters	Δρ_max_ = 0.26 e Å^−^^3^
6 restraints	Δρ_min_ = −0.29 e Å^−^^3^

### Special details

**Table:**

Geometry. All esds (except the esd in the dihedral angle between two l.s. planes) are estimated using the full covariance matrix. The cell esds are taken into account individually in the estimation of esds in distances, angles and torsion angles; correlations between esds in cell parameters are only used when they are defined by crystal symmetry. An approximate (isotropic) treatment of cell esds is used for estimating esds involving l.s. planes.
Refinement. Refinement of F^2^ against ALL reflections. The weighted R-factor wR and goodness of fit S are based on F^2^, conventional R-factors R are based on F, with F set to zero for negative F^2^. The threshold expression of F^2^ > 2sigma(F^2^) is used only for calculating R-factors(gt) etc. and is not relevant to the choice of reflections for refinement. R-factors based on F^2^ are statistically about twice as large as those based on F, and R- factors based on ALL data will be even larger.

### Fractional atomic coordinates and isotropic or equivalent isotropic displacement parameters (Å^2^)

**Table:**

	*x*	*y*	*z*	*U*_iso_*/*U*_eq_	
Mn1	0.86083 (5)	0.64881 (3)	0.57766 (4)	0.03184 (15)	
O1	0.7990 (3)	0.77011 (13)	0.48474 (19)	0.0610 (8)	
O2	0.9562 (3)	0.76311 (12)	0.63126 (18)	0.0498 (7)	
O3	1.0696 (2)	1.00516 (13)	0.85451 (17)	0.0443 (6)	
O4	1.0246 (3)	1.11522 (13)	0.77017 (18)	0.0507 (7)	
O5	0.6696 (3)	1.04731 (14)	0.43566 (19)	0.0708 (9)	
N1	0.6456 (3)	0.66681 (15)	0.6502 (2)	0.0391 (7)	
N2	0.6681 (3)	0.60012 (15)	0.4629 (2)	0.0337 (6)	
C1	0.8656 (4)	0.88801 (18)	0.5761 (3)	0.0330 (8)	
C2	0.9443 (3)	0.92408 (18)	0.6647 (2)	0.0332 (8)	
H2	1.0049	0.8952	0.7155	0.040*	
C3	0.9327 (3)	1.00281 (18)	0.6774 (2)	0.0310 (7)	
C4	0.8419 (4)	1.04587 (19)	0.6018 (3)	0.0418 (9)	
H4	0.8343	1.0989	0.6102	0.050*	
C5	0.7631 (4)	1.0102 (2)	0.5143 (3)	0.0453 (9)	
C6	0.7755 (4)	0.93092 (18)	0.5017 (3)	0.0417 (9)	
H6	0.7225	0.9068	0.4425	0.050*	
C7	0.8744 (4)	0.80229 (19)	0.5621 (3)	0.0402 (9)	
C8	1.0148 (3)	1.0427 (2)	0.7738 (3)	0.0348 (8)	
C9	0.6513 (6)	1.1283 (2)	0.4467 (3)	0.0872 (17)	
H9A	0.6116	1.1389	0.5127	0.131*	
H9B	0.5824	1.1473	0.3878	0.131*	
H9C	0.7475	1.1532	0.4471	0.131*	
C10	0.6399 (4)	0.7003 (2)	0.7453 (3)	0.0499 (10)	
H10	0.7309	0.7132	0.7860	0.060*	
C11	0.5089 (4)	0.7168 (2)	0.7862 (3)	0.0554 (10)	
H11	0.5107	0.7407	0.8526	0.067*	
C12	0.3748 (4)	0.6973 (2)	0.7273 (3)	0.0607 (11)	
H12	0.2835	0.7073	0.7533	0.073*	
C13	0.3765 (4)	0.6624 (2)	0.6285 (3)	0.0527 (10)	
H13	0.2862	0.6492	0.5872	0.063*	
C14	0.5131 (3)	0.64735 (19)	0.5918 (2)	0.0348 (8)	
C15	0.5263 (3)	0.60952 (18)	0.4878 (2)	0.0317 (8)	
C16	0.4026 (4)	0.5843 (2)	0.4195 (3)	0.0468 (9)	
H16	0.3056	0.5903	0.4383	0.056*	
C17	0.4231 (4)	0.5504 (2)	0.3238 (3)	0.0532 (10)	
H17	0.3403	0.5339	0.2769	0.064*	
C18	0.5661 (4)	0.5414 (2)	0.2983 (3)	0.0523 (10)	
H18	0.5829	0.5187	0.2338	0.063*	
C19	0.6858 (4)	0.5666 (2)	0.3703 (3)	0.0457 (9)	
H19	0.7836	0.5598	0.3531	0.055*	
O6	1.0261 (3)	0.62274 (13)	0.47247 (18)	0.0421 (6)	
H1W	1.037 (4)	0.6603 (12)	0.432 (2)	0.063*	
H2W	1.033 (4)	0.5804 (11)	0.440 (2)	0.063*	
O7	0.0671 (3)	0.74055 (16)	0.3430 (2)	0.0584 (7)	
H3W	0.041 (4)	0.734 (2)	0.2772 (16)	0.088*	
H4W	0.016 (4)	0.7774 (18)	0.364 (3)	0.088*	

### Atomic displacement parameters (Å^2^)

**Table:**

	*U*^11^	*U*^22^	*U*^33^	*U*^12^	*U*^13^	*U*^23^
Mn1	0.0319 (3)	0.0319 (3)	0.0307 (3)	−0.0004 (2)	0.0002 (2)	−0.0032 (2)
O1	0.098 (2)	0.0369 (15)	0.0428 (15)	−0.0028 (14)	−0.0116 (15)	−0.0114 (13)
O2	0.0714 (17)	0.0300 (14)	0.0447 (15)	0.0034 (12)	−0.0057 (13)	0.0006 (12)
O3	0.0495 (15)	0.0450 (15)	0.0357 (14)	−0.0025 (12)	−0.0049 (11)	0.0000 (12)
O4	0.0683 (17)	0.0278 (14)	0.0521 (16)	−0.0073 (12)	−0.0081 (13)	−0.0043 (12)
O5	0.107 (2)	0.0441 (16)	0.0505 (16)	0.0136 (16)	−0.0334 (16)	0.0026 (14)
N1	0.0396 (16)	0.0476 (18)	0.0297 (16)	0.0028 (14)	0.0037 (13)	−0.0038 (14)
N2	0.0332 (15)	0.0381 (17)	0.0299 (15)	−0.0007 (13)	0.0046 (12)	−0.0070 (13)
C1	0.047 (2)	0.0278 (17)	0.0244 (17)	−0.0025 (16)	0.0053 (15)	−0.0002 (15)
C2	0.0362 (19)	0.033 (2)	0.0303 (19)	−0.0003 (15)	0.0046 (15)	0.0053 (15)
C3	0.0375 (18)	0.0296 (19)	0.0264 (18)	−0.0064 (15)	0.0063 (14)	−0.0025 (15)
C4	0.058 (2)	0.0267 (18)	0.039 (2)	0.0013 (17)	−0.0033 (18)	−0.0013 (16)
C5	0.062 (2)	0.039 (2)	0.032 (2)	0.0034 (18)	−0.0067 (18)	0.0023 (17)
C6	0.060 (2)	0.030 (2)	0.032 (2)	−0.0023 (17)	−0.0035 (18)	−0.0043 (16)
C7	0.056 (2)	0.033 (2)	0.033 (2)	−0.0039 (18)	0.0117 (18)	−0.0007 (17)
C8	0.0336 (19)	0.036 (2)	0.036 (2)	−0.0019 (16)	0.0068 (16)	−0.0061 (17)
C9	0.140 (5)	0.044 (3)	0.065 (3)	0.029 (3)	−0.036 (3)	0.000 (2)
C10	0.050 (2)	0.066 (3)	0.034 (2)	−0.001 (2)	0.0065 (18)	−0.0099 (19)
C11	0.065 (3)	0.066 (3)	0.037 (2)	0.013 (2)	0.017 (2)	−0.006 (2)
C12	0.050 (2)	0.079 (3)	0.058 (3)	0.015 (2)	0.024 (2)	0.000 (2)
C13	0.037 (2)	0.071 (3)	0.050 (2)	0.006 (2)	0.0069 (17)	−0.004 (2)
C14	0.0328 (17)	0.0364 (19)	0.0352 (19)	0.0041 (16)	0.0035 (15)	0.0046 (17)
C15	0.0284 (18)	0.0344 (19)	0.0323 (19)	0.0017 (14)	0.0035 (14)	0.0018 (15)
C16	0.0317 (19)	0.067 (3)	0.041 (2)	−0.0044 (18)	0.0006 (16)	−0.007 (2)
C17	0.047 (2)	0.069 (3)	0.041 (2)	−0.015 (2)	−0.0054 (18)	−0.011 (2)
C18	0.050 (2)	0.068 (3)	0.038 (2)	−0.006 (2)	0.0030 (19)	−0.016 (2)
C19	0.038 (2)	0.060 (3)	0.040 (2)	0.0013 (18)	0.0044 (17)	−0.0136 (19)
O6	0.0435 (14)	0.0394 (14)	0.0444 (16)	−0.0025 (13)	0.0090 (12)	−0.0006 (12)
O7	0.0654 (19)	0.0565 (19)	0.0529 (17)	0.0004 (14)	0.0060 (15)	0.0046 (15)

### Geometric parameters (Å, °)

**Table:**

Mn1—O4^i^	2.135 (2)	C5—C6	1.392 (4)
Mn1—O6	2.147 (2)	C6—H6	0.9300
Mn1—O2	2.230 (2)	C9—H9A	0.9600
Mn1—N1	2.246 (3)	C9—H9B	0.9600
Mn1—N2	2.264 (2)	C9—H9C	0.9600
Mn1—O1	2.439 (2)	C10—C11	1.364 (5)
O1—C7	1.244 (4)	C10—H10	0.9300
O2—C7	1.261 (4)	C11—C12	1.368 (5)
O3—C8	1.254 (4)	C11—H11	0.9300
O4—C8	1.263 (4)	C12—C13	1.384 (5)
O4—Mn1^ii^	2.135 (2)	C12—H12	0.9300
O5—C5	1.372 (4)	C13—C14	1.379 (4)
O5—C9	1.424 (4)	C13—H13	0.9300
N1—C10	1.337 (4)	C14—C15	1.482 (4)
N1—C14	1.353 (4)	C15—C16	1.381 (4)
N2—C19	1.330 (4)	C16—C17	1.373 (5)
N2—C15	1.349 (4)	C16—H16	0.9300
C1—C6	1.375 (4)	C17—C18	1.361 (5)
C1—C2	1.389 (4)	C17—H17	0.9300
C1—C7	1.502 (4)	C18—C19	1.381 (4)
C2—C3	1.382 (4)	C18—H18	0.9300
C2—H2	0.9300	C19—H19	0.9300
C3—C4	1.388 (4)	O6—H1W	0.837 (17)
C3—C8	1.504 (4)	O6—H2W	0.846 (17)
C4—C5	1.376 (4)	O7—H3W	0.839 (18)
C4—H4	0.9300	O7—H4W	0.847 (18)
			
O4^i^—Mn1—O6	101.98 (9)	O2—C7—C1	119.2 (3)
O4^i^—Mn1—O2	81.42 (9)	O3—C8—O4	121.7 (3)
O6—Mn1—O2	96.29 (9)	O3—C8—C3	120.9 (3)
O4^i^—Mn1—N1	90.59 (9)	O4—C8—C3	117.4 (3)
O6—Mn1—N1	164.94 (9)	O5—C9—H9A	109.5
O2—Mn1—N1	93.75 (9)	O5—C9—H9B	109.5
O4^i^—Mn1—N2	135.16 (10)	H9A—C9—H9B	109.5
O6—Mn1—N2	92.96 (9)	O5—C9—H9C	109.5
O2—Mn1—N2	139.02 (9)	H9A—C9—H9C	109.5
N1—Mn1—N2	72.15 (9)	H9B—C9—H9C	109.5
O4^i^—Mn1—O1	136.04 (9)	N1—C10—C11	124.0 (3)
O6—Mn1—O1	91.03 (9)	N1—C10—H10	118.0
O2—Mn1—O1	55.30 (8)	C11—C10—H10	118.0
N1—Mn1—O1	85.48 (9)	C10—C11—C12	118.3 (3)
N2—Mn1—O1	84.78 (9)	C10—C11—H11	120.9
C7—O1—Mn1	86.9 (2)	C12—C11—H11	120.9
C7—O2—Mn1	96.11 (19)	C11—C12—C13	119.2 (4)
C8—O4—Mn1^ii^	105.5 (2)	C11—C12—H12	120.4
C5—O5—C9	117.4 (3)	C13—C12—H12	120.4
C10—N1—C14	117.8 (3)	C14—C13—C12	119.5 (3)
C10—N1—Mn1	123.6 (2)	C14—C13—H13	120.2
C14—N1—Mn1	118.4 (2)	C12—C13—H13	120.2
C19—N2—C15	118.3 (3)	N1—C14—C13	121.1 (3)
C19—N2—Mn1	124.0 (2)	N1—C14—C15	115.5 (3)
C15—N2—Mn1	117.7 (2)	C13—C14—C15	123.4 (3)
C6—C1—C2	119.7 (3)	N2—C15—C16	121.0 (3)
C6—C1—C7	119.5 (3)	N2—C15—C14	116.0 (3)
C2—C1—C7	120.8 (3)	C16—C15—C14	123.0 (3)
C3—C2—C1	120.1 (3)	C17—C16—C15	119.9 (3)
C3—C2—H2	119.9	C17—C16—H16	120.0
C1—C2—H2	119.9	C15—C16—H16	120.0
C2—C3—C4	119.9 (3)	C18—C17—C16	119.2 (3)
C2—C3—C8	120.9 (3)	C18—C17—H17	120.4
C4—C3—C8	119.1 (3)	C16—C17—H17	120.4
C5—C4—C3	120.1 (3)	C17—C18—C19	118.5 (3)
C5—C4—H4	120.0	C17—C18—H18	120.7
C3—C4—H4	120.0	C19—C18—H18	120.7
O5—C5—C4	124.6 (3)	N2—C19—C18	123.2 (3)
O5—C5—C6	115.6 (3)	N2—C19—H19	118.4
C4—C5—C6	119.8 (3)	C18—C19—H19	118.4
C1—C6—C5	120.4 (3)	Mn1—O6—H1W	110 (2)
C1—C6—H6	119.8	Mn1—O6—H2W	125 (2)
C5—C6—H6	119.8	H1W—O6—H2W	112 (3)
O1—C7—O2	120.4 (3)	H3W—O7—H4W	108 (3)
O1—C7—C1	120.4 (3)		
			
O4^i^—Mn1—O1—C7	5.0 (3)	O5—C5—C6—C1	−179.7 (3)
O6—Mn1—O1—C7	−103.5 (2)	C4—C5—C6—C1	0.2 (5)
O2—Mn1—O1—C7	−6.56 (19)	Mn1—O1—C7—O2	11.1 (3)
N1—Mn1—O1—C7	91.2 (2)	Mn1—O1—C7—C1	−167.1 (3)
N2—Mn1—O1—C7	163.6 (2)	Mn1—O2—C7—O1	−12.2 (4)
O4^i^—Mn1—O2—C7	−165.4 (2)	Mn1—O2—C7—C1	166.0 (3)
O6—Mn1—O2—C7	93.4 (2)	C6—C1—C7—O1	−1.1 (5)
N1—Mn1—O2—C7	−75.4 (2)	C2—C1—C7—O1	177.2 (3)
N2—Mn1—O2—C7	−8.5 (3)	C6—C1—C7—O2	−179.3 (3)
O1—Mn1—O2—C7	6.50 (19)	C2—C1—C7—O2	−1.0 (5)
O4^i^—Mn1—N1—C10	41.7 (3)	Mn1^ii^—O4—C8—O3	−1.6 (4)
O6—Mn1—N1—C10	−171.5 (3)	Mn1^ii^—O4—C8—C3	177.8 (2)
O2—Mn1—N1—C10	−39.8 (3)	C2—C3—C8—O3	−14.7 (5)
N2—Mn1—N1—C10	179.6 (3)	C4—C3—C8—O3	164.0 (3)
O1—Mn1—N1—C10	−94.5 (3)	C2—C3—C8—O4	166.0 (3)
O4^i^—Mn1—N1—C14	−142.8 (2)	C4—C3—C8—O4	−15.4 (4)
O6—Mn1—N1—C14	4.0 (5)	C14—N1—C10—C11	−0.8 (5)
O2—Mn1—N1—C14	135.8 (2)	Mn1—N1—C10—C11	174.8 (3)
N2—Mn1—N1—C14	−4.9 (2)	N1—C10—C11—C12	0.7 (6)
O1—Mn1—N1—C14	81.0 (2)	C10—C11—C12—C13	−0.6 (6)
O4^i^—Mn1—N2—C19	−107.3 (3)	C11—C12—C13—C14	0.6 (6)
O6—Mn1—N2—C19	3.0 (3)	C10—N1—C14—C13	0.8 (5)
O2—Mn1—N2—C19	106.1 (3)	Mn1—N1—C14—C13	−175.0 (3)
N1—Mn1—N2—C19	−179.3 (3)	C10—N1—C14—C15	−179.2 (3)
O1—Mn1—N2—C19	93.7 (3)	Mn1—N1—C14—C15	5.0 (4)
O4^i^—Mn1—N2—C15	76.3 (3)	C12—C13—C14—N1	−0.7 (5)
O6—Mn1—N2—C15	−173.4 (2)	C12—C13—C14—C15	179.3 (3)
O2—Mn1—N2—C15	−70.3 (3)	C19—N2—C15—C16	0.6 (5)
N1—Mn1—N2—C15	4.3 (2)	Mn1—N2—C15—C16	177.2 (2)
O1—Mn1—N2—C15	−82.6 (2)	C19—N2—C15—C14	−179.9 (3)
C6—C1—C2—C3	−0.4 (5)	Mn1—N2—C15—C14	−3.3 (4)
C7—C1—C2—C3	−178.7 (3)	N1—C14—C15—N2	−1.0 (4)
C1—C2—C3—C4	0.2 (5)	C13—C14—C15—N2	178.9 (3)
C1—C2—C3—C8	178.9 (3)	N1—C14—C15—C16	178.4 (3)
C2—C3—C4—C5	0.2 (5)	C13—C14—C15—C16	−1.6 (5)
C8—C3—C4—C5	−178.5 (3)	N2—C15—C16—C17	−1.2 (5)
C9—O5—C5—C4	−1.1 (6)	C14—C15—C16—C17	179.3 (3)
C9—O5—C5—C6	178.7 (4)	C15—C16—C17—C18	0.8 (6)
C3—C4—C5—O5	179.5 (3)	C16—C17—C18—C19	0.1 (6)
C3—C4—C5—C6	−0.4 (5)	C15—N2—C19—C18	0.4 (5)
C2—C1—C6—C5	0.2 (5)	Mn1—N2—C19—C18	−176.0 (3)
C7—C1—C6—C5	178.6 (3)	C17—C18—C19—N2	−0.8 (6)

Symmetry codes: (i) −*x*+2, *y*−1/2, −*z*+3/2; (ii) −*x*+2, *y*+1/2, −*z*+3/2.

### Hydrogen-bond geometry (Å, °)

**Table:**

*D*—H···*A*	*D*—H	H···*A*	*D*···*A*	*D*—H···*A*
O6—H1W···O7^iii^	0.84 (2)	1.83 (2)	2.668 (4)	178 (4)
O6—H2W···O3^iv^	0.85 (2)	1.89 (2)	2.726 (3)	171 (3)
O7—H3W···O2^v^	0.84 (2)	1.90 (2)	2.724 (3)	169 (4)

Symmetry codes: (iii) *x*+1, *y*, *z*; (iv) *x*, −*y*+3/2, *z*−1/2; (v) *x*−1, −*y*+3/2, *z*−1/2.
